# Critical amino acid residues in human ACE2 for SARS-CoV-2 spike protein binding and virus entry

**DOI:** 10.1128/spectrum.03244-24

**Published:** 2025-06-20

**Authors:** Weiyi Chen, Joo-Youn Lee, Jae-Sung Kim, Jin Soo Shin, To Sing Fung, Jung-Yong Yeh, Zhenhai Chen, Bin Zhou, Ji-Joon Song, Yun Young Go

**Affiliations:** 1 Department of Infectious Diseases and Public Health, Jockey Club College of Veterinary Medicine and Life Sciences, City University of Hong Kong, Hong Kong SAR, China; 2 Therapeutics and Biotechnology Division, Korea Research Institute of Chemical Technology 65680, Daejeon, Republic of Korea; 3 Department of Biological Sciences, Korea Advanced Institute of Science and Technology 124657 , Daejeon, Republic of Korea; 4 Department of Life Sciences, College of Life Sciences and Bioengineering, Incheon National University 665558, Incheon, Republic of Korea; 5 College of Veterinary Medicine, Yangzhou University 614704, Yangzhou, China; 6 MOE Joint International Research Laboratory of Animal Health and Food Safety, College of Veterinary Medicine, Nanjing Agricultural University 261674, Nanjing, China; 7 College of Veterinary Medicine, Konkuk University, Seoul, Republic of Korea; Shandong First Medical University, Jinan, Shandong, China

**Keywords:** SARS-CoV-2, S-ACE2 interaction, ACE2 variants, amino acid substitutions, virus entry, binding affinity, syncytium formation, molecular dynamics simulation

## Abstract

The coronavirus disease 2019 (COVID-19) pandemic, caused by severe acute respiratory syndrome coronavirus-2 (SARS-CoV-2), remains a significant global public health concern due to the continuous emergence and rapid spread of new variants. SARS-CoV-2 utilizes angiotensin-converting enzyme 2 (ACE2) as its primary receptor to initiate viral entry into host cells. While ACE2 is highly conserved across different species, genetic variability in the interacting surfaces between ACE2 orthologs and SARS-CoV-2 spike (S) protein can modulate viral binding affinity and entry efficiency. This study investigates the impact of amino acid substitutions in human ACE2 (hACE2) interacting with the receptor-binding domain of SARS-CoV-2 S protein. Site-directed mutagenesis, combined with molecular dynamics simulations and pseudovirus assays, revealed that D30V and H34R substitutions reduce hACE2 binding affinity and fusogenic activity, impairing SARS-CoV-2 entry. However, the double mutant D30V-H34R did not reduce viral entry efficiency further, suggesting compensatory molecular interactions at the ACE2-S binding interface. These insights contribute to a deeper understanding of SARS-CoV-2-host interactions and may guide future therapeutic development targeting viral entry mechanisms.

**IMPORTANCE:**

Given the pivotal role of angiotensin-converting enzyme 2 (ACE2) in mediating viral entry and the genetic divergence observed in ACE2 orthologs across different species, we aimed to elucidate further the molecular intricacies underlying the interactions between severe acute respiratory syndrome coronavirus-2 (SARS-CoV-2) spike (S) protein and ACE2. In this study, we examined the amino acid residues in ACE2 orthologs interacting with SARS-CoV-2 spike receptor-binding domain to identify those with discernible effects on viral binding and entry. Through *in vitro* mutagenesis and *in silico* modeling studies of ACE2 variants, we have pinpointed the amino acid substitutions in human ACE2 that affect SARS-CoV-2 binding and entry. This work can significantly advance our understanding of the molecular mechanisms of SARS-CoV-2-host interactions, receptor recognition, viral entry process, and potential therapeutic options targeting coronavirus entry.

## INTRODUCTION

The coronavirus disease 2019 (COVID-19) pandemic, driven by severe acute respiratory syndrome coronavirus-2 (SARS-CoV-2), has profoundly impacted global health and socio-economic systems. SARS-CoV-2 is the third highly pathogenic coronavirus of the *Coronaviridae* family that emerged in the past two decades following outbreaks of SARS-CoV in 2003 and Middle East respiratory syndrome coronavirus (MERS-CoV) in 2013 (1–3). Despite the World Health Organization declaration ending COVID-19 as a global health emergency in May 2023, SARS-CoV-2 remains a persistent public health threat, with recurring infections fueled by new variants capable of immune escape, rendering current vaccines and therapeutic antibodies less effective (4–9).

Zoonotic coronaviruses emerge following a transmission event between reservoir species and a new susceptible host, where viral and host factors, such as viral surface protein-receptor interactions and host proteases, support productive virus infection (10, 11). SARS-CoV-2 utilizes the spike (S) glycoprotein on its surface to engage with angiotensin-converting enzyme 2 (ACE2), the primary receptor for viral entry into host cells (12). The S protein is composed of two subunits: the S1 and S2. The S1 subunit is responsible for receptor recognition of target cell surface via its receptor-binding domain (RBD). The S2 subunit comprises the homotrimeric spike core, facilitating membrane fusion upon viral binding (13). During viral infection, the S protein is cleaved into S1 and S2 subunits, facilitating conformational changes and exposing the S2′ cleavage site within the S2 subunit after viral binding to the cellular receptors (14, 15). Subsequently, cleavage at the S2′ site is needed to release an internal fusion peptide and trigger the interaction between the heptad repeat HR1 and HR2 domains in the S2 subunit, forming a six-helix bundle fusion core that brings the viral and cellular membranes closely for fusion (16–18).

ACE2 is widely expressed in various animal species, including the presumed natural reservoirs and intermediate hosts of SARS-CoV-2, such as bats, pangolins, domestic animals, and feral carnivores (19–23). Given the zoonotic nature of SARS-CoV-2 infection, numerous investigations have been conducted to study the interactions between SARS-CoV-2 S protein and ACE2 orthologs found in vertebrates, aiming to explore natural hosts, cross-species transmission, experimental animal models, and molecular basis for SARS-CoV-2 entry (24–26). ACE2 is conserved among vertebrates, with varying expression levels in tissues such as the lungs, gastrointestinal tract, and cardiovascular system (27, 28). However, polymorphisms in ACE2 have been reported across and within species, affecting the ability of SARS-CoV-2 to bind and enter host cells (29–31). Therefore, the intermolecular interactions between ACE2-RBD complexes have been extensively investigated to identify potential amino acid residues pivotal for SARS-CoV-2 entry (22, 24, 26, 32, 33). Specific amino acid residues within ACE2 orthologs, particularly at positions 30, 34, hotspot-31, and hotspot-353 within the ACE2-RBD binding interface, have been shown to play a crucial role in determining SARS-CoV-2 binding patterns across different species (33–39). Substitutions at these sites can disrupt interactions with the viral S protein, reducing binding affinity and impairing viral entry into host cells (25, 33, 35, 40). Similarly, single nucleotide polymorphisms in human ACE2 (hACE2), such as rs73635825 (S19P) and rs143936283 (E329G) located at the SARS-CoV-2 S-ACE2 interface, were reported to affect the interaction with the SARS-CoV-2 S protein, thus influencing virus entry and varying disease outcomes in the human population (41–43). Considering the pivotal role of ACE2 in mediating virus entry and the genetic divergence observed in ACE2 orthologs across different species, we aimed to elucidate the molecular intricacies underlying the interaction between SARS-CoV-2 S protein and ACE2. In this study, we have experimentally evaluated the interactions between the wild-type SARS-CoV-2 S protein (Wuhan-Hu-1 strain) and ACE2 orthologs from multiple species with different susceptibilities to identify potential amino acid residues with discernible effects on virus binding and entry. Using a combination of mutagenesis study, molecular dynamics (MD) simulations, and pseudovirus entry assays, we determined that amino acid changes at D30 and H34 affect the interaction of hACE2 with SARS-CoV-2 S protein. These findings offer additional molecular insights into host ACE2-S interactions, providing guidance for the development of therapeutic interventions targeting viral entry.

## MATERIALS AND METHODS

### Cells and antibodies

Human embryonic kidney 293T cells (HEK293T cells) from the American Type Culture Collection (ATCC; Manassas, VA, USA) were cultured in Dulbecco’s modified Eagle medium (DMEM; Gibco, Waltham, MA, USA) supplemented with 10% fetal bovine serum (FBS; Gibco), 10 mM 4-(2-hydroxyethyl)-1-piperazine ethanesulfonic acid (HEPES; Gibco), and 1% penicillin-streptomycin (Gibco) at 37℃ in a 5% CO_2_ incubator. Baby hamster kidney cells (BHK21 cells; ATCC) were cultured in DMEM containing 10% FBS.

Rabbit anti-SARS-CoV-2 S protein antibody was purchased from Sino Biological Inc. (Beijing, China). 6X His-tag mouse monoclonal antibody and goat anti-mouse IgG (H + L) secondary antibody (Alexa Fluor 488) were purchased from Invitrogen (Waltham, MA, USA). Alexa Fluor 647-conjugated rabbit IgG antibody was purchased from Abcam (Cambridge, UK).

### Construction of ACE2 and S protein expression plasmids

The full-length ACE2 coding sequences of human (*Homo sapiens*, GQ262784.1), cattle (*Bos taurus*, BT021667), cat (*Feline catus*, NM_001039456), civet (*Paguma larvata*, AY881174), dog (*Canis lupus familiaris*, NM_001165260), ferret (*Mustela putorius furo*, NM_001310190), hamster (*Mesocricetus auratus*, XP_005074266), mouse (*Mus musculus*, BC026801), pig (*Sus scrofa*, EU518378), and rat (*Rattus norvegicus*, GQ262788) were synthesized by Beijing Genomic Institute (BGI, Beijing, China) and cloned into a pcDNA3.1/myc-His A expression vector (Invitrogen) between *NotI* and *KpnI* sites to obtain pcDNA3.1/myc-His-ACE2 expression plasmids (Table S1). The ACE2 protein expression and transfection efficiency were checked using immunofluorescence staining (Fig. S1).

The S gene expression plasmid (cat no. VG40589; Sino Biological Inc.) encoding the wild-type SARS-CoV-2 spike protein (Wuhan-Hu-1 strain) with silent mutations to optimize expression in mammalian cells was used as a template for PCR amplification. The translated amino acid sequence is identical to the original SARS-CoV-2 Wuhan-Hu-1 strain (GenBank: MN908947.3). The amplified full-length S gene was cloned into pcDNA3.1 (+) vector (Invitrogen) between *KpnI* and *XbaI* restriction sites to obtain pcDNA3.1-SARS2-S plasmid. In addition, the pcDNA3.1-SARS2-S served as a template to amplify the S protein with 18 amino acid deletions in the C-terminus, which was utilized to generate the pcDNA3.1-SARS2-S-Δ18 plasmid used in producing high titered SARS-CoV-2 pseudovirus particles.

### Site-directed mutagenesis of hACE2-expressing plasmids

Single amino acid mutations (D30-I/L/N/V, K31E, H34-K/L/Q/R/Y, D38-F/I/L/V, or K353H) were introduced into the pcDNA3.1/myc-His-hACE2 plasmid using QuikChange II XL Site-Directed Mutagenesis Kit (Agilent Technologies, Santa Clara, CA, USA) according to the manufacturer’s instructions (Table S2). After performing mutant strand synthesis using *PfuUltra* high-fidelity (HF) DNA polymerase with oligonucleotide primers containing desired mutations, parental DNA templates were digested with *DpnI*, and the remaining PCR product was transformed into competent cells to obtain clones with desired mutations. The mutations were verified by DNA sequence analysis from BGI, and their expression level was checked by immunofluorescence assay (Fig. S2).

### Syncytium formation assay

Approximately 80%–90% confluent HEK293T cells in 12-well plates were transfected with pcDNA3.1/*myc*-His-ACE2 or pcDNA3.1-SARS2-S plasmid using Lipofectamine 2000 (Invitrogen). After 24 h of incubation, cells expressing ACE2 and SARS-CoV-2 S were mixed at a 1:1 ratio and incubated in a 5% CO_2_ incubator at 37°C. After 6 h of incubation, cells were fixed in the 4% paraformaldehyde solution, and multinucleated syncytium formation was assessed by immunofluorescence assay. Briefly, fixed cells were permeabilized using 0.2% Triton X-100 (Invitrogen) in 1× Dulbecco’s phosphate-buffered saline (DPBS) for 10 min and blocked with 5% FBS in 1× DPBS for 1 h at room temperature (RT). Subsequently, cells were incubated with anti-SARS-CoV-2 S antibody and 6X His-tag antibody at RT for 1 h, followed by incubation with Alexa Fluor 488-conjugated anti-mouse IgG and Alexa Fluor 647-conjugated anti-rabbit IgG secondary antibodies for 1 h in a dark environment. After washing with 1× DPBS, cells were mounted in SlowFade Diamond Antifade Mountant (Invitrogen) with 4′,6-diamidino-2-phenylindole (DAPI, Invitrogen), and fluorescence images were recorded using ECLIPSE Ti2 inverted fluorescence microscope (Nikon, Melville, NY, USA). Nine imaging fields of multinucleated syncytia were randomly chosen for each ACE2 variant across three independent experiments, and their surface area was measured using NIS-Elements image analysis software (Nikon). The fusogenic activity was calculated as (μS_variant_ − μS_cell_)/(μS_WT_ − μS_cell_) × 100%, where μS_cell_ is the average area of unfused single HEK293T cells, μS_variant_ is the average fusion area formed by indicated ACE2 variant, and μS_WT_ is the average fusion area formed by wild-type hACE2.

### SARS-CoV-2-S pseudovirus production and quantification

As previously described, pseudotyped SARS-CoV-2 particles with a firefly luciferase (FLuc) reporter were produced in HEK293T cells (44, 45). Briefly, cells in a 100 mm cell culture dish were co-transfected with 10 µg of pLVXS-FLuc (Takara Bio, San Jose, CA, USA), 10 µg of psPAX2 (Addgene, Watertown, MA, USA), and 5 µg of pcDNA3.1-SARS2-S-Δ18 using Lipofectamine 2000, following the manufacturer’s protocols. After 72–96 h of transfection, the supernatant containing pseudotyped SARS-CoV-2 was harvested and concentrated using a PEG Precipitation Kit (Abcam). Subsequently, the pseudotyped virus was titrated with a Lenti-X qRT-PCR Titration Kit (Takara), aliquoted, and stored at −80°C for further use. The known copy numbers of the Lenti-X RNA control template from the kit were used to generate standard curves, and the viral copy number was calculated accordingly. Finally, the pseudovirus input volume was adjusted to the same titer (copies/mL) for the following pseudovirus-based infectivity assay.

### Pseudovirus entry assay

The pseudovirus entry assay was performed using a similar approach as reported elsewhere (44, 46). In brief, BHK21 cells were transfected with plasmids expressing ACE2 of different animal species or hACE2 variants using Lipofectamine 2000 following the standard protocol. At 24 h post-transfection, cells were washed with PBS and infected with pseudotyped SARS-CoV-2 at approximately 2 × 10^7^ copies/mL. At 48 h post-infection, the supernatant was removed, and the cells were lysed with 20 µL of 1× firefly luciferase lysis buffer (Promega, Madison, WI, USA). Cell lysates were mixed with firefly luciferase substrates, and the luciferase signals were immediately measured by a SpectraMax iD3 Multi-Mode Microplate Reader (Molecular Devices, Sunnyvale, CA, USA). The luciferase activity was expressed as the number of relative light units (RLU). The pseudovirus entry rate was quantified as (μRLU_variant_ − μRLU_mock_)/(μRLU_WT_ − μRLU_mock_) × 100%, where μRLU_mock_ is the average RLU from non-transfected BHK21 cells infected with the pseudotyped SARS-CoV-2, μRLU_variant_ is the average RLU from BHK21 cells expressing indicated ACE2 variants infected with SARS-CoV-2 pseudovirus, and μRLU_WT_ is the average RLU from BHK21 cells expressing wild-type hACE2 infected with SARS-CoV-2 pseudovirus. Each condition was performed in triplicate across three independent experiments.

### Surface plasmon resonance binding analysis of ACE2-RBD

The binding kinetics and affinity of SARS-CoV-2 S RBD with hACE2 variants containing D30V or H34R mutations were determined using Biacore T200 (Cytiva, Marlborough, MA, USA). SARS-CoV-2 S RBD was immobilized to a CM5 sensor chip (Cytiva) and a running buffer of 150 mM NaCl and 20 mM HEPES (pH = 7.4). Then, twofold serial dilutions of wild-type or mutant hACE2 were flowed through with a concentration ranging from 400 to 0 nM. The real-time association and dissociation kinetics of the RBD-ACE2 interaction were monitored, and the response data were analyzed and fitted with a 1:1 binding model using Biacore software.

### MD simulation

The SARS-CoV-2 S-ACE2 complex models with D30V and H34R mutations in hACE2 were generated using the residue mutation tool from the Schrödinger Maestro Suite (Schrödinger, LLC, New York, NY, USA, 2020), which employed the X-ray structure of the SARS-CoV-2 S protein in complex with human ACE2 (PDB code 6LZG) as the template. The complexes were then prepared using the Protein Preparation Wizard, and the Desmond v6.1 (Desmond Molecular Dynamics System; D. E. Shaw Research: New York, NY, USA) as well as the OPLS3e force field from the Schrödinger Suite software were employed for the MD simulations. The System Builder was used for solvation, employing predefined TIP3P water molecules in an orthorhombic box with dimensions of 12 Å × 12 Å × 12 Å (47), and the overall complex was neutralized by adding Cl^−^ counterions. The NaCl salt concentration was 0.15 mol/L. Production of MD simulations at 150 ns in length was carried out under periodic boundary conditions in the isothermal-isobaric (NPT) ensemble at normal temperatures (300 K) and pressure (1.01325 bar). Recording intervals of 1.2 and 15 ps were used for energy calculation and trajectory analysis. The analyzed protein interface and proposed interaction model of SARS-CoV-2/ACE2 for the final frame of the MD simulations were represented using Discovery Studio 2018 (Dassault Systèmes Biovia, San Diego, CA, USA).

### Statistics analysis

Data were analyzed using Student’s *t*-test or one-way analysis of variance (ANOVA) followed by Dunnett’s multiple comparisons in GraphPad Prism (GraphPad Software Inc., San Diego, CA, USA). Statistical analyses were expressed as the mean ± standard deviation (SD). Differences were considered statistically significant when the *P* value was <0.05.

## RESULTS

### Functional evaluation of ACE2 orthologs in SARS-CoV-2 fusion efficiency

Although ACE2 is conserved across species, host susceptibility to SARS-CoV-2 infection varies. To explore the potential determinants of this variability affecting viral entry, we first comparatively analyzed the amino acid sequences of 10 ACE2 orthologs from species with documented susceptibility or resistance to SARS-CoV-2 based on experimental or natural infection studies. These included highly susceptible species, such as humans, cats, hamsters, and ferrets; species with reported infections in experimental and natural settings, such as dogs and cattle; and non-susceptible species, including civets, pigs, mice, and rats. The sequence alignment revealed a high degree of conservation among the examined ACE2 orthologs, with sequence identities ranging from 81.4% (pig) to 85.2% (cat) compared to hACE2 (Table S3). These species shared identical sequences in most contact residues from the SARS-CoV-2 S-ACE2 protein virus-binding motif. However, specific residues, such as D30, K31, H34, and D38, showed variability, potentially influencing receptor usage and viral susceptibility.

**Fig 1:**
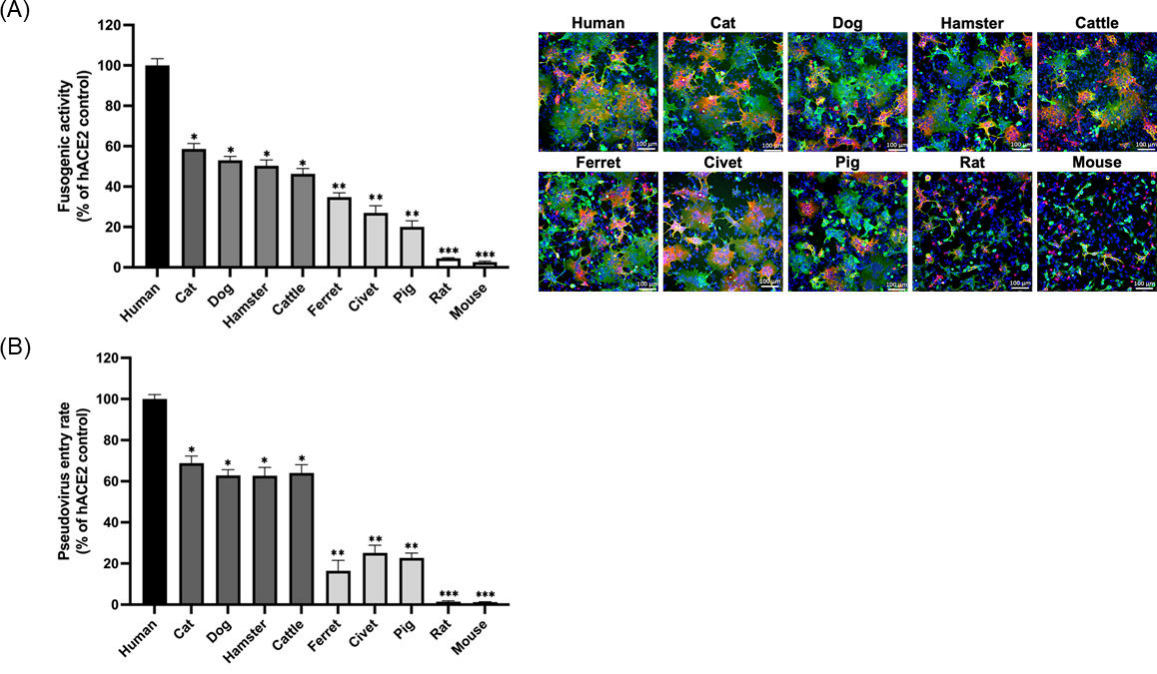
Assessment of ACE2 receptor usage by SARS-CoV-2 in different animal species. (**A**) HEK293T cells transfected with plasmids expressing different animal ACE2s (human/cat/dog/cattle/ferret/civet/pig/hamster/mouse/rat) were mixed with cells transfected with a plasmid expressing full-length S protein of SARS-CoV-2 at a 1:1 ratio. After 6 h of incubation, cells were fixed, and syncytium formation was stained with antibodies to detect ACE2 protein (green), spike protein (red), and DAPI (blue, nuclei). Nine fusion fields were randomly chosen for each species, and the surface areas were measured using Nikon measurement software and then compared to hACE2 (considered as 100%). Representative pictures of syncytium formation are shown in the right panel (scale bar: 100 µm). (**B**) BHK21 cells expressing different animal ACE2s were transduced with SARS-CoV-2 pseudovirus-bearing S protein and a firefly luciferase reporter gene. At 48 h post-infection, luciferase activity was measured and compared to that of hACE2 (considered as 100%). Data are presented as mean ± SD from three independent experiments. For the syncytium formation assay, quantification was based on nine imaging fields for each ACE2 variant derived from three independent experiments. For the pseudovirus entry assay, measurements were obtained from three independent experiments performed in triplicate. Statistical significance was assessed using one-way ANOVA followed by Dunnett’s multiple comparisons and unpaired *t*-test. Significance levels are indicated by *P < 0.05, **P < 0.01, and ***P < 0.001.

Given the high sequence similarity among ACE2 orthologs, it is challenging to differentiate receptor usage and host susceptibilities among various species solely based on comparative sequence analysis. To further assess how variations in the ACE2 receptor affect its fusogenic activity, we conducted a syncytium formation assay by co-culturing HEK293T cells expressing ACE2 orthologs with cells expressing SARS-CoV-2 S protein. The fusion efficiency was quantified by measuring the surface area of multinucleated syncytia (Fig. 1A). Compared to wild-type hACE2, which was assigned 100% fusogenic activity, the ACE2 orthologs from cats (57.6% ± 2.8%), dogs (53.0% ± 2.1%), hamsters (50.3% ± 2.9%), and cattle (46.3% ± 2.7%) showed comparable sizes of syncytia to each other, but smaller than those of hACE2. In contrast, ferrets (34.8% ± 1.9%), civets (27.0% ± 3.5%), and pigs (20.1% ± 3.1%) exhibited significantly lower fusion efficiency compared to those of cats, dogs, hamsters, and cattle ACE2-expressing cells. As expected, mice and rats resistant to SARS-CoV-2 displayed minimal fusogenic activity, with syncytial areas of 2.6% ± 0.5% and 4.5% ± 0.3%, respectively (Fig. 1A). These results confirm that while ACE2 sequence similarity is generally predictive of receptor functionality, certain amino acid residues might significantly impact the efficiency of SARS-CoV-2 attachment and membrane fusion.

### Differential efficiency of ACE2 orthologs in mediating SARS-CoV-2 pseudovirus entry across species

To further validate the different capacities of ACE2 orthologs in facilitating SARS-CoV-2 entry, pseudovirus assays were conducted using SARS-CoV-2 pseudoparticles encoding a firefly luciferase reporter gene. ACE2 orthologs from 10 different species were transiently expressed in BHK21 cells, and luciferase activity was measured 48 h post-infection to assess SARS-CoV-2 entry efficiency. The pseudovirus entry efficiency for each ACE2 ortholog was compared to that of hACE2-expressing cells, which was set as 100%. The results were aligned with the findings from the syncytium formation assay (Fig. 1B). Cells expressing cat (68.8% ± 3.5%), cattle (64.0% ± 4.1%), dog (62.8% ± 2.9%), and hamster (62.7% ± 4.1%) ACE2s exhibited comparable pseudovirus entry efficiency, confirming their role in facilitating SARS-CoV-2 entry. Conversely, ACE2 orthologs from civets (25.1% ± 3.7%) and pigs (22.7% ± 2.3%) displayed lower viral entry efficiencies, consistent with their previously observed reduced fusogenic activity. Ferret ACE2 supported limited viral entry (16.5% ± 5.1%) despite the species being highly susceptible to SARS-CoV-2 *in vivo*, warranting further investigation. As anticipated, ACE2 orthologs from mice (1.1% ± 0.3%) and rats (1.9% ± 0.5%) failed to mediate efficient SARS-CoV-2 entry, correlating with their resistance to infection. Together, these results highlight the role of ACE2 orthologs in modulating SARS-CoV-2 entry and underscore the complexity of receptor usage across species.

### Functional analysis of key residues in hACE2 critical for SARS-CoV-2 syncytium formation

**Fig 2:**
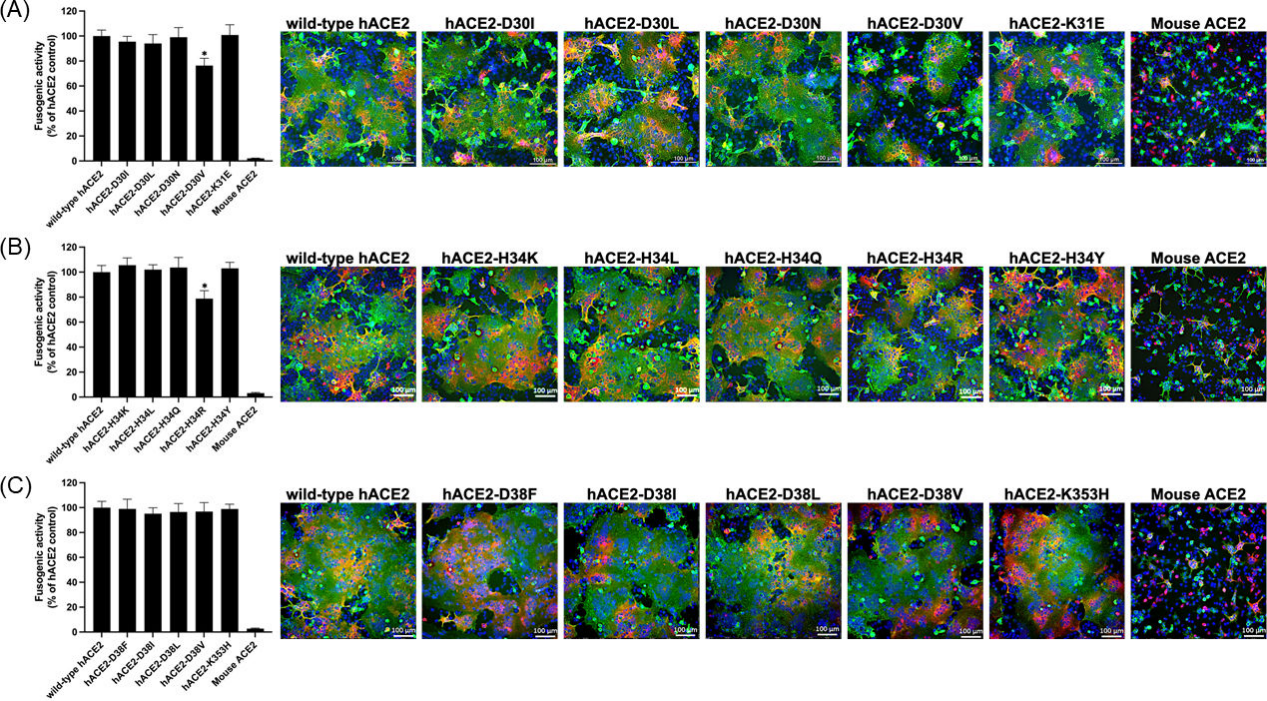
Identification of critical amino acid substitutions in hACE2 interacting with SARS-CoV-2 S protein. Syncytia formed by hACE2 containing different mutations (**A**) D30I/L/N/V and K31E, (**B**) H34K/L/Q/R/Y, and (**C**) D38F/I/L/V and K353H were fixed and stained with anti-ACE2 protein antibody (green), anti-S protein antibody (red), and DAPI (blue, nuclei). Nine surface areas of syncytium formation were measured among different mutant hACE2s using Nikon measurement software and compared to wild-type hACE2 (WT) (considered as 100%). Mouse ACE2 was included as a negative control. Data are presented as mean ± SD from three independent experiments of nine randomly selected imaging fields. Statistical significance was assessed using one-way ANOVA followed by Dunnett’s multiple comparisons. Significance levels are indicated by *P < 0.05. Representative pictures with a scale bar of 100 µm are shown in the right panel.

To elucidate the functional importance of specific amino acid residues in hACE2 for SARS-CoV-2 recognition and membrane fusion, we introduced site-specific mutations at positions D30, H34, hotspot-31 (K31), and hotspot-353 (D38 and K353). These residues were selected based on structural insights from published literature (33–39) and comparative analysis across species, which we further substantiated using MD simulations (Fig. S3; Table 1). Our findings indicated the functional significance of residues such as D30 in human and hamster ACE2, which forms a salt bridge with K417 in the RBD, a critical interaction disrupted by the N30 substitution in mice and rats. Similarly, H34 (human and cat) and Y34 (ferret) maintain hydrophobic interactions with L455 in the RBD, whereas substitutions like L34 or Q34 in other species abolish this interaction, potentially reducing receptor binding efficiency. In hotspot-31, K31 interacts with E484 in the RBD across most studied species, while substitutions like T31 in civets and N31 in mice compromise this interaction. Hotspot-353 also plays an important role in receptor recognition, yet becomes unstable in mouse and rat ACE2 due to the H353 substitution. Furthermore, D38 in hACE2 forms hydrogen bonds with Q493 and S494 in the RBD, but these interactions are notably absent in cattle, hamster, pig, mouse, and rat ACE2, indicating a significant variability in ACE2-RBD stability among species (Fig. S3; Table 1). Together with previous studies, these molecular variations suggested that the amino acid residues at D30, H34, hotspot-31, and hotspot-353 could influence the molecular interactions with SARS-CoV-2 RBD, thereby potentially altering host receptor binding properties across different species. To assess the functional implications of these amino acid variations, we conducted quantitative syncytium formation assays using HEK293T cells expressing hACE2 variants with mutations at the aforementioned positions. Briefly, substitutions at D30, including isoleucine (I), leucine (L), asparagine (N), and valine (V), as well as K31 to glutamic acid (E), were designed to investigate the effects of variations in charge, polarity, hydrophobicity, and side-chain size on hACE2’s interaction with the SARS-CoV-2 S protein and its fusogenic activity (Fig. 2A). While D30I, D30L, D30N, and K31E substitutions induced syncytia of similar size to those observed with wild-type hACE2, the D30V substitution significantly impaired fusogenic activity, with a fusion efficiency of 76.3% ± 5.8% relative to wild-type hACE2. Similarly, substitutions at H34 (H34L, H34Q, H34K, H34Y, and H34R) were tested to evaluate the impact of varying physicochemical properties on residue function. Among these, only the H34R mutation resulted in a notable reduction in fusion efficiency (78.8% ± 6.3%) compared to wild-type hACE2, while other substitutions had no significant impact (Fig. 2B). In contrast, mutations at D38 (D38F, D38I, D38L, and D38V) and K353 (K353H) showed no effect on syncytial formation, indicating that these are less critical for hACE2-mediated fusion (Fig. 2C). These results highlight the critical roles of D30 and H34 in facilitating the interaction between hACE2 and the SARS-CoV-2 S protein. Specific substitutions, particularly D30V and H34R, appear to disrupt this interaction, reducing fusion efficiency and potentially impairing viral entry.

**TABLE 1:** Comparison of potential critical ACE2 residues of different animal species that affect binding to SARS-CoV-2 RBD

SARS-CoV-2 RBD	Human ACE2	Human ACE2/RBD interaction	Cattle ACE2	Cat ACE2	Civet ACE2	Dog ACE2	Ferret ACE2	Hamster ACE2	Pig ACE2	Mouse ACE2	Rat ACE2
K417	D30	Electrostatic	E30	E30	E30	E29	E30	–	E30	N30	N30
E484	K31*^a^*	Electrostatic	–^*c*^	–	T31	–	–	–	–	N31	–
L455	H34	Hydrophobic	–	–	Y34	Y33	Y34	Q34	L34	Q34	Q34
Q493/S494	D38*^b^*	Hydrogen bond	–	E38	E38	E37	E38	–	–	–	–
G502	K353*^b^*	Hydrogen bond	–	–	–	–	–	–	–	H353	H353

^*a*^ Hotspot-31: formation of an intramolecular interaction between K31 and E35 (salt bridge).

^*b*^ Hotspot-353: formation of an intramolecular interaction between K353 and D38 (salt bridge).

^*c*^ – denotes no amino acid change in the position compared to the human ACE2 sequence.

### Impact of D30V and H34R substitutions on SARS-CoV-2 entry and ACE2-RBD binding affinity

**Fig 3:**
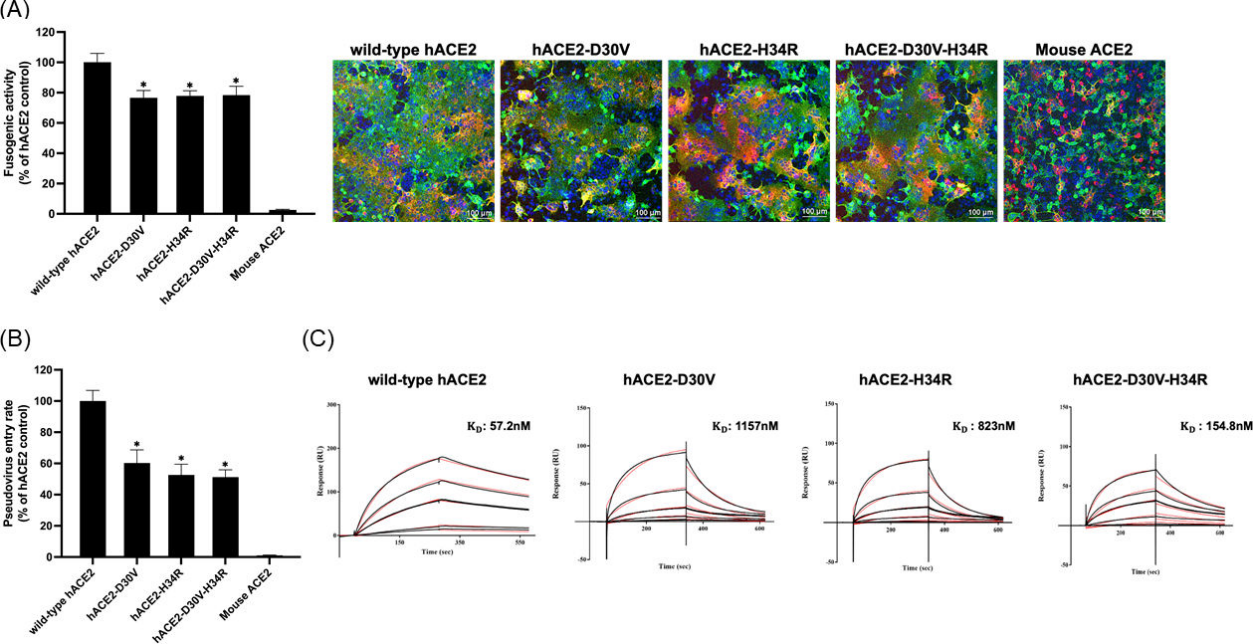
Investigation of D30V and H34R substitutions in hACE2 that negatively affect SARS-CoV-2 binding and entry. (**A**) Syncytium formation assay of wild-type hACE2, single mutant hACE2 D30V, H34R, and double mutant hACE2 D30V-H34R along with mouse as the negative control was performed and stained with anti-ACE2 protein antibody (green), anti-S protein antibody (red), and DAPI (blue, nuclei). Scale bar: 100 µm. The fusion areas were measured and compared to hACE2 (WT) using Nikon measurement software. (**B**) BHK21 cells expressing wild-type or mutant hACE2 proteins (mouse ACE2 as negative control) were infected by SARS-CoV-2 pseudovirus carrying S protein and a firefly luciferase reporter gene. At 48 h post-infection, luciferase activity was measured and compared to hACE2 (WT) (considered as 100%). (**C**) The binding kinetics (Kd) of wild-type and mutant hACE2 with SARS-CoV-2 RBD were obtained using Biacore. SARS-CoV-2 RBD was immobilized to the sensor chip, and serial dilutions of hACE2 variants were then injected over the chip surface. One set of representative data is displayed. Data are presented as mean ± SD from three independent experiments. For the syncytium formation assay, quantification was based on nine imaging fields for each ACE2 variant derived from three independent experiments. For the pseudovirus entry assay, measurements were obtained from three independent experiments performed in triplicate. Statistical significance was assessed using one-way ANOVA followed by Dunnett’s multiple comparisons and unpaired *t*-test. Significance levels are indicated by *P < 0.05.

To assess the effects of the D30V and H34R substitutions in hACE2 on receptor-mediated SARS-CoV-2 entry and binding to the viral RBD, syncytium formation assays, pseudovirus entry assays, and surface plasmon resonance (SPR) binding analyses were performed. The D30V and H34R mutations were introduced individually and in combination (D30V-H34R double mutant) to investigate their impact on membrane fusion, viral entry, and ACE2-RBD binding affinity. In syncytium formation assays, cells expressing either the D30V or H34R mutant showed significantly reduced fusion activity compared to wild-type hACE2, with fusion efficiencies of 76.5% ± 4.8% and 77.8% ± 3.4%, respectively (Fig. 3A). Interestingly, the D30V-H34R double mutant did not exacerbate the reduction in fusion efficiency, yielding a fusion efficiency of 78.2% ± 5.8%, suggesting potential compensatory interactions between the two mutations. Similarly, in pseudovirus entry assays, cells expressing D30V or H34R ACE2 mutants exhibited marked reductions in viral entry efficiency, with decreases of 41% and 47%, respectively, compared to wild-type hACE2 (Fig. 3B). The D30V-H34R double mutant showed no additional inhibition, with a 49% reduction in entry efficiency, consistent with the syncytium formation results. To confirm the direct effect of these substitutions on ACE2-RBD binding, SPR binding analysis was performed using the SARS-CoV-2 RBD immobilized on a sensor chip. Wild-type hACE2 displayed a strong binding affinity with a dissociation constant (Kd) of 57.2 nM. In contrast, the D30V and H34R mutants showed significantly weaker binding affinities, with Kd values of 1,157 nM and 823 nM, respectively (Fig. 3C). The double mutant exhibited an intermediate binding reduction, with a Kd of 154.8 nM, suggesting that compensatory molecular interactions between the mutations mitigate the impact of the individual alterations. These results demonstrate that D30V and H34R substitutions impair SARS-CoV-2 entry by disrupting ACE2-RBD interactions, though compensatory effects between these residues may partially preserve receptor functionality in the double mutant.

### Molecular mechanisms of D30V and H34R substitutions on ACE2-RBD interactions

**Fig 4:**
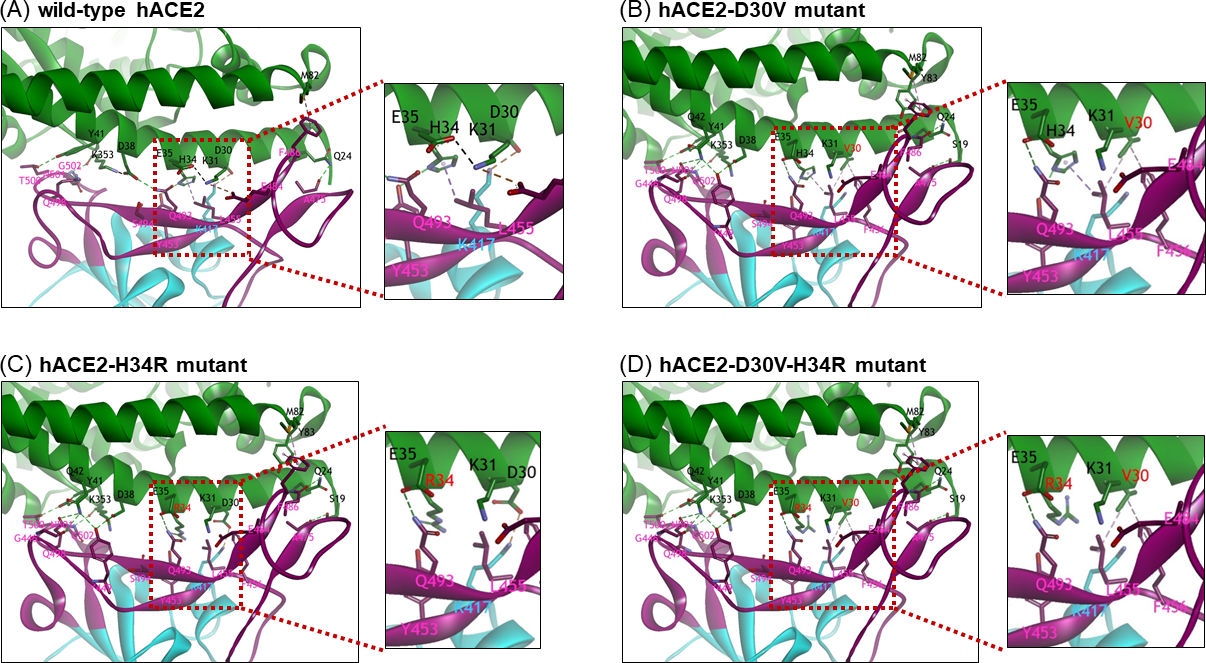
Molecular interaction of human ACE2 mutants complexed with SARS-CoV-2 RBD. MD simulations illustrate the interactions between the SARS-CoV-2 RBD and (**A**) wild-type hACE2, (**B**) hACE2 D30V mutant, (**C**) hACE2 H34R mutant, and (**D**) hACE2 D30V-H34R double mutant. The hACE2 structure is shown in green, with mutations highlighted in red, while the RBD region in the S protein of SARS-CoV-2 is shown in magenta. The right inset panels provide magnified views of the red-boxed regions in the left panels, showing the detailed residue-specific ACE2-RBD interactions affected by the D30V and H34R mutations. The hydrogen bonds are shown as green dashed lines, and hydrophobic and electrostatic interactions are represented by pink and orange dashed lines, respectively. Black dashed lines display the intramolecular interactions within hACE2.

To investigate the molecular basis for the impact of the D30V and H34R substitutions on ACE2 functionality, homology-based structural modeling and molecular dynamics simulations were employed. Using the wild-type hACE2-RBD complex as a template, the structural effects of the D30V or H34R mutations were analyzed to uncover the changes in molecular interactions at the interface between hACE2 and the SARS-CoV-2 spike RBD. The D30V substitution disrupted the strong electrostatic interaction between D30 and K417 in the spike RBD. Instead, this mutation introduced weaker hydrophobic contacts with L455 and F456, reducing the stability of the ACE2-RBD interface (Fig. 4A and B). Similarly, the H34R substitution abolished hydrophobic interactions with L455 and disrupted a hydrogen bond with Y453 of the spike RBD, further destabilizing the ACE2-RBD complex (Fig. 4C). Interestingly, in the D30V-H34R double mutant, the structural alterations caused by the V30 substitution shortened the distance between R34 and L455. While R34 failed to establish hydrophobic interactions with L455, the closer proximity facilitated electrostatic stabilization, partially compensating for the destabilizing effects of the individual mutations (Fig. 4D). These findings indicate that the single D30V or H34R mutation impairs ACE2-RBD binding and destabilizes the complex through distinct mechanisms. However, the structural changes induced by the double mutant lead to compensatory interactions that mitigate the individual deficits, explaining the observed moderate reductions in binding and fusion efficiency. This structural insight highlights the intricate molecular interplay at the ACE2-RBD interface.

## DISCUSSION

The transmission and pathogenesis of SARS-CoV-2 are critically influenced by host-virus interactions, with ACE2 serving as the primary receptor for viral entry. Despite the high sequence conservation among ACE2 orthologs, subtle amino acid variations at key binding residues can significantly alter interactions with the SARS-CoV-2 S protein, thereby modulating receptor functionality and host susceptibility (29, 37, 48–50). In this study, we examined the functional roles of ACE2 orthologs across species with varying susceptibility to SARS-CoV-2 infection. Consistent with their high susceptibility to natural infections, ACE2 orthologs from cats and hamsters exhibited robust receptor functionality, facilitating efficient pseudovirus entry (51, 52). Similarly, cattle and dog ACE2s supported efficient SARS-CoV-2 entry, corroborating previous experimental and natural infection data (53–55). In contrast, ACE2 orthologs from civets, pigs, mice, and rats displayed reduced receptor activity, aligning with their resistance to SARS-CoV-2 infection (22, 48, 56–58). Intriguingly, despite being highly susceptible *in vivo*, ferrets showed only modest ACE2-mediated fusion and pseudovirus entry *in vitro*. Similar to our results, others also reported minimal binding activity between ferret ACE2 and the SARS-CoV-2 S1 protein, along with limited pseudovirus entry (33) (48). This discrepancy may be attributed to factors beyond ACE2, such as ACE2 expression patterns and co-factors like TMPRSS2, which significantly enhance ACE2-mediated fusion in ferrets (48, 59, 60).

The specific amino acid composition of ACE2 at the binding interface critically determines its interaction with the SARS-CoV-2 S protein, influencing viral entry and host susceptibility. However, due to the high sequence similarity among ACE2 orthologs, distinguishing receptor usage and susceptibility across different species can be challenging. To address this, we combined mutagenesis studies with MD simulations to gain a more comprehensive understanding of the binding and structural dynamics of the ACE2-RBD interaction across different species and in different hACE2 mutants. Our findings revealed that D30V and H34R substitutions in hACE2 significantly reduce its functionality, as evidenced by diminished fusogenic activity, reduced pseudovirus entry efficiency, and weaker ACE2-RBD binding affinity. These results align with previous studies highlighting the importance of residues 30 and 34 in the ACE2-S interface. D30 in wild-type hACE2, for example, forms a crucial salt bridge with K417 in the RBD, a key electrostatic interaction absent in non-susceptible species like mice and rats (N30) (61–63). While the D30V mutation has not yet been observed in any animal species or ACE2 polymorphisms, its occurrence could potentially influence hACE2 binding and fusogenic activity. Similarly, H34 contributes essential hydrophobic interactions with the SARS-CoV-2 RBD. Only H34 in human and cat ACE2 or Y34 in ferret ACE2 can form these bonds (38, 64–66), while L34 of pigs or Q34 of mice and rats disrupt these interactions, correlating with their resistance to SARS-CoV-2 infection (35). In particular, the H34R mutation disrupts polar contacts with the SARS-CoV-2 RBD, providing insights into the role of ACE2 polymorphisms in influencing viral entry, as shown in genomic studies (31). Furthermore, Li *et al.* demonstrated that the H34R mutation in horseshoe bat ACE2 significantly inhibited pseudovirus entry of the SARS-CoV-2-related bat coronavirus RaTG13 by interfering with hydrogen bonding to Y453 and disrupting van der Waals and hydrophobic interactions with L455 (67). These findings underscore the critical roles of amino acid residues 30 and 34 in ACE2 functionality and suggest that species-specific differences in these residues can profoundly influence SARS-CoV-2 entry.

Interestingly, our study demonstrated that while both D30V and H34R substitutions individually impair ACE2-RBD binding and SARS-CoV entry, their combined effect in the D30V-H34R double mutant did not further reduce viral entry efficiency. To gain deeper insight into this phenomenon, MD simulations were conducted to capture the natural motion and conformational changes of the ACE2–RBD complex under varying conditions. During the COVID-19 pandemic, MD simulations have become a widely used computational approach for investigating dynamic virus-host interactions, particularly those involving the SARS-CoV-2 S protein and ACE2 receptor (38, 68, 69). In our study, MD simulations allowed for atomic-level analysis of the stability and conformational dynamics at the ACE2-RBD interface, offering mechanistic insights that static structural models or conventional experimental approaches alone cannot provide. The simulations revealed that the D30V substitution disrupts the electrostatic interaction with K417 but compensates by introducing new hydrophobic interactions with L455 and F456. Conversely, the H34R substitution eliminates hydrophobic interactions with L455 but allows electrostatic stabilization in the presence of the D30V mutation. These compensatory effects at the ACE2-RBD interface mitigate the overall impact of the double mutant, highlighting the complexity of protein-protein interactions at a molecular level. The insights gained from MD simulations have important implications for understanding ACE2-SARS-CoV-2 interactions and potential host susceptibility, particularly in elucidating the molecular mechanisms underlying viral infection in evolving SARS-CoV-2 variants.

SARS-CoV-2 has undergone rapid evolution, leading to the emergence of variants like Alpha (lineage B.1.1.7), Beta (lineage B.1.351), Gamma (lineage P1), Delta (lineage B.1.617.2), and the current Omicron sub-lineages, each harboring distinct mutations in the spike protein that critically impact ACE2 binding (70, 71). Key mutations within the RBD, including E484K and N501Y in the Beta and Gamma variants and L452R in the Delta variant, have been demonstrated to enhance the binding affinity of ACE2, facilitating more efficient viral infection (72–74). Furthermore, mutations in the C-terminal domain of the S1 subunit, such as D614G, H655Y, and P681H/R, present in several SARS-CoV-2 variants, are known to increase viral fusogenicity and promote viral entry, thereby contributing to the adaptability and infectivity of SARS-CoV-2 (75–77). Notably, mutations at K417, such as K417T (Gamma variant) and K417N (Beta and Omicron variants), disrupt the critical salt bridge with D30 in ACE2, leading to a reduction in ACE2 binding affinity (78, 79). However, this effect is often mitigated by compensatory mutations, such as N501Y and D614G in the Beta, Gamma, and Omicron variants, which enhance the overall binding affinity of the spike protein to ACE2, stabilizing the ACE2-S interface and restoring viral fitness (73, 75, 80). Such compensatory mechanisms are crucial for cross-species transmission and were pivotal in the adaptation of SARS-CoV-2 to infect previously non-susceptible hosts like mice (73, 81, 82). Moreover, the emergence of mutations such as L455S in the circulating Omicron sub-lineage JN.1 has been associated with enhanced viral entry efficiency *in vitro*, likely due to a stronger interaction with H34 of ACE2 (83). Notably, these residue-specific interactions differ among sarbecoviruses. For instance, bat coronavirus RaTG13 forms weaker interactions with D30 and H34 in hACE2, reducing binding affinity for hACE2 compared to SARS-CoV-2 (84, 85). These adaptive mutations underscore the complex interplay between spike protein variability and ACE2 polymorphisms, potentially altering susceptibility profiles and affecting viral entry efficiency across different hosts (71, 78).

From a therapeutic perspective, targeting the ACE2-S interface remains a promising strategy for blocking viral entry. Engineered peptide mimics or soluble ACE2 derivatives could effectively neutralize the virus by competing with host receptors (86–88). While our study focused on *in vitro* experiments, further validation of these findings involving infectious viruses *in vivo* is needed. Additionally, the role of other host factors, such as the serine protease TMPRSS2, which primes the spike protein for fusion, warrants further investigation (12, 48). Moreover, the potential involvement of alternative receptors, such as CD209L and CD147, in facilitating viral entry may offer new opportunities for therapeutic development (89, 90). Thus, understanding how multiple receptors contribute to viral entry could provide novel insights into SARS-CoV-2 pathogenesis and guide the development of broad-spectrum antiviral strategies.

In conclusion, our study highlights the critical roles of D30 and H34 in hACE2 functionality and demonstrates that compensatory structural changes can mitigate the impact of specific amino acid substitutions. These findings enhance our understanding of the molecular determinants of SARS-CoV-2 entry and provide valuable insights for developing antiviral strategies targeting viral-receptor interactions.

## SUPPLEMENTAL MATERIAL

Supplemental material spectrum.03244-24-s0001.docx Figures S1 to S3 and Tables S1 to S3.

ASM does not own the copyrights to Supplemental Material that may be linked to, or accessed through, an article. The authors have granted ASM a non-exclusive, world-wide license to publish the Supplemental Material files. Please contact the corresponding author directly for reuse.
