# Supplementary material for: Critical amino acid residues in human ACE2 for SARS-CoV-2 spike protein binding and virus entry
Source: Microbiol Spectr. 2025 Jun 20;13(8):e03244-24. doi: 10.1128/spectrum.03244-24 (PMC12323325; doi:10.1128/spectrum.03244-24)
Supplement: Supplemental material — Figures S1 to S3 and Tables S1 to S3. [file spectrum.03244-24-s0001.docx]

**Supplementary Material**

**Supplementary Figure S1.**


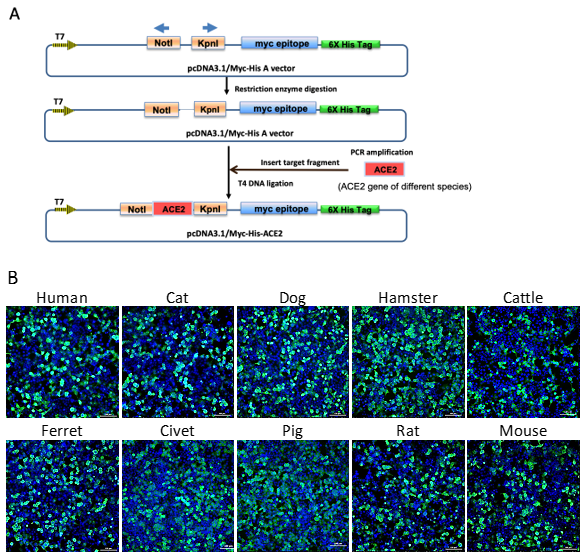


**Figure S1.** Construction and expression of ACE2 gene from different species. (A) Schematic diagram of ACE2 gene cloning into pcDNA 3.1/myc-His A vector. (B) Identification of different animal ACE2 genes expression through immunofluorescence assay. The plasmids pcDNA3.1/myc-His-ACE2 containing ACE2 gene of different species (human, cat, dog, hamster, cattle, ferret, civet, pig, rat, and mouse) were transfected into HEK293T cells. After 24 hours of incubation, cells were fixed, and immunofluorescence staining was performed and recorded using Nikon inverted microscope (ACE2 protein (Green), DAPI (Blue, nuclei), Scale bars: 100 μm).

**Supplementary Figure S2.**


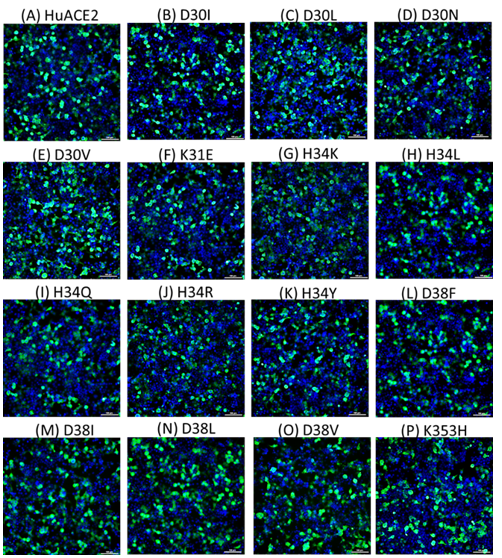


**Figure S2.** Transfection efficiency of mutant huACE2s with different mutations. The plasmids containing different mutant huACE2 genes (A) wild-type huACE2, (B) D30I, (C) D30L, (D) D30N, (E) D30V, (F) K31E, (G) H34K, (H) H34L, (I) H34Q, (J) H34R, (K) H34Y, (L) D38F, (M) D38I, (N) D38L, (O) D38V, and (P) K353H were transfected into HEK293T cells. Cells were fixed after 24 hours of incubation, and immunofluorescence staining was performed and recorded using Nikon inverted microscope (ACE2 protein (Green), DAPI (Blue, nuclei), Scale bars: 100 μm).

**Supplementary Figure S3.**


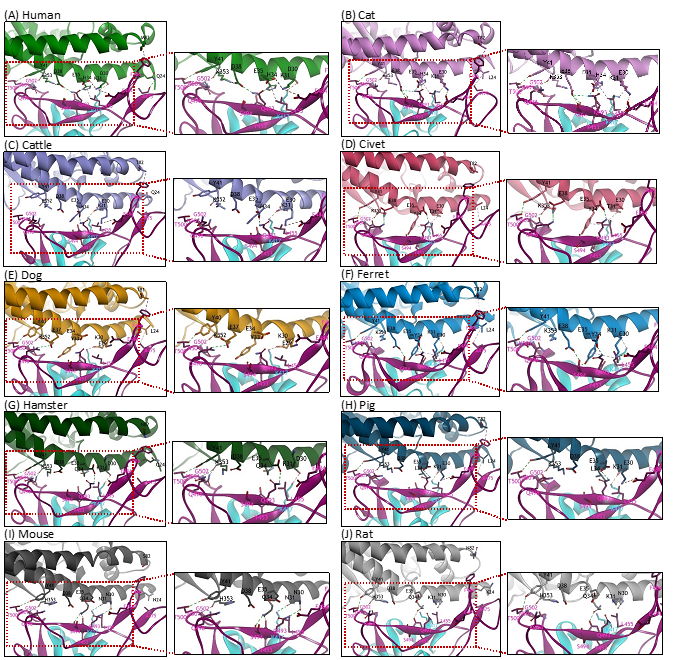


**Figure S3.** Analyses of protein binding interface on molecular dynamics simulation of SARS-CoV-2 S protein with ACE2 from various animals. (A) human ACE2 (green), (B) cat ACE2 (pink), (C) cattle ACE2 (purple), (D) civet ACE2 (fuchsia), (E) dog ACE2 (orange), (F) ferret ACE2 (blue), (G) hamster (dark green), (H) pig ACE2 (dark blue), (I) mouse ACE2 (black), and (G) rat ACE2 (grey) complexed with S protein are shown. The RBD region in the S protein of SARS-CoV-2 is shown in magenta. The right inset panels provide magnified views of the red-boxed regions in the left panels, showing the detailed residue-specific ACE2-RBD interactions at D30, H34, hotspot-31, and hotspot-353 in different species. The hydrogen bonds are shown as green dashed lines and hydrophobic and electrostatic interactions are represented by pink and orange dashed lines, respectively.

**Table S1** ACE2 and SARS-CoV-2 expressing plasmids and primers used for cloning.

| **Gene name** | **Recombinant plasmid** | **Primer name** | **Primer sequence (5'-3')** |
| --- | --- | --- | --- |
| Human ACE2 | pcDNA3.1/myc-His-HuACE2 | HuACE2-NotI-F | AAATATGCGGCCGCGCCACCATGTCAAGCTCTTCCTGGCTC |
|  |  | HuACE2-KpnI-R | CGGGGTACCAAAGGAGGTCTGAACATCATCAG |
| Cat ACE2 | pcDNA3.1/myc-His-CatACE2 | CatACE2-NotI-F | AAATATGCGGCCGCGCCACCATGTCAGGCTCTTTCTGGCTC |
|  |  | CatACE2-KpnI-R | CGGGGTACCAAATGAAGTCTGAACATCATCAGC |
| Cattle ACE2 | pcDNA3.1/myc-His-CattleACE2 | CattleACE2-NotI-F | AAATATGCGGCCGCGCCACCATGACAGGCTCTTTCTGGCTC |
|  |  | CattleACE2-KpnI-R | CGGGGTACCAAGCGAAGTCTGAACATCATCA |
| Civet ACE2 | pcDNA3.1/myc-His-CivetACE2 | CivetACE2-NotI-F | AAATATGCGGCCGCGCCACCATGTCAGGCTCTTTCTGGCT |
|  |  | CivetACE2-KpnI-R | CGGGGTACCAAATGAAGTCTGAACGTCA |
| Dog ACE2 | pcDNA3.1/myc-His-DogACE2 | DogACE2-NotI-F | AAATATGCGGCCGCGCCACCATGTCAGGCTCTTCCTGGCTC |
|  |  | DogACE2-KpnI-R | CGGGGTACCAAACGAAGTCTGAACATCATCAC |
| Ferret ACE2 | pcDNA3.1/myc-His-FerretACE2 | FerretACE2-NotI-F | AAATATGCGGCCGCGCCACCATGTTAGGCTCTTCCTGGCTC |
|  |  | FerretACE2-KpnI-R | CGGGGTACCAAATGACGTCTGAACATCATCAACA |
| Hamster ACE2 | pcDNA3.1/myc-His-HamsterACE2 | HamsterACE2-NotI-F | AAATATGCGGCCGCGCCACCATGTCAAGCTCCTCCTGGCTC |
|  |  | HamsterACE2-KpnI-R | CGGGGTACCAAATGAAGTCTGAGCATCATCA |
| Pig ACE2 | pcDNA3.1/myc-His-PigACE2 | PigACE2-NotI-F | AAATATGCGGCCGCGCCACCATGTCAGGCTCTTTCTGGCTC |
|  |  | PigACE2-KpnI-R | CGGGGTACCAAACGAAGTCTGAATGTCATCGC |
| Mouse ACE2 | pcDNA3.1/myc-His-MouseACE2 | MouseACE2-NotI-F | AAATATGCGGCCGCGCCACCATGTCCAGCTCCTCCTGGC |
|  |  | MouseACE2-KpnI-R | CGGGGTACCAAAGGAAGTCTGAGCATCATC |
| Rat ACE2 | pcDNA3.1/myc-His-RatACE2 | RatACE2-NotI-F | AAATATGCGGCCGCGCCACCATGTCAAGCTCCTGCTGGC |
|  |  | RatACE2-KpnI-R | CGGGGTACCGAATGAAGTTTGAGCATCATCA |
| SARS-CoV-2 spike protein | pcDNA3.1-SARS2-S | SARS2-S-KpnI-F | CGGGGTACCGCCACCATGTTTGTGTTCCTGGTGCTGC |
|  |  | SARS2-S-XbaI-R | GCTCTAGATCAGGTGTAGTGCAGTTTCACTC |
| SARS-CoV-2 spike protein with 18 amino acids deletion | pcDNA3.1-SARS2-S-Δ18 | BamHI-SARS2-S-M1-F | AGAGGATCCACCATGTTTGTGTTCCTGGTGC |
|  |  | SARS2-S-K1255-XhoI-R | GAGACTCGAGTCACTTACAACAGGAGCCACAGGAAC |

**Table S2** Amino acid mutations introduced into wild-type huACE2 and primers used for site-directed mutagenesis.

| **Mutation in huACE2** | **Recombinant plasmid** | **Primer name** | **Primer sequence (5'-3')** |
| --- | --- | --- | --- |
| HuACE2 D30-I | pcDNA3.1-HuACE2-D30I | HuACE2_D30I-F | CGGCTTCGTGGTTAAACTTGATCAAAAATGTCTTGGCCTGTT |
|  |  | HuACE2_D30I-R | AACAGGCCAAGACATTTTTGATCAAGTTTAACCACGAAGCCG |
| HuACE2 D30-L | pcDNA3.1-HuACE2-D30L | HuACE2_D30L-F | CTTCGGCTTCGTGGTTAAACTTTAGCAAAAATGTCTTGGCCTGTTCC |
|  |  | HuACE2_D30L-R | GGAACAGGCCAAGACATTTTTGCTAAAGTTTAACCACGAAGCCGAAG |
| HuACE2 D30-N | pcDNA3.1-HuACE2-D30N | HuACE2_D30N-F | GCTTCGTGGTTAAACTTGTTCAAAAATGTCTTGGCCTGT |
|  |  | HuACE2_D30N-R | ACAGGCCAAGACATTTTTGAACAAGTTTAACCACGAAGC |
| HuACE2 D30-V | pcDNA3.1-HuACE2-D30V | HuACE2_D30V-F | GCTTCGTGGTTAAACTTGACCAAAAATGTCTTGGCCT |
|  |  | HuACE2_D30V-R | AGGCCAAGACATTTTTGGTCAAGTTTAACCACGAAGC |
| HuACE2 K31-E | pcDNA3.1-HuACE2-K31E | HuACE2_K31E-F | CGGCTTCGTGGTTAAACTCGTCCAAAAATGTCTTGGC |
|  |  | HuACE2_K31E-R | GCCAAGACATTTTTGGACGAGTTTAACCACGAAGCCG |
| HuACE2 H34-K | pcDNA3.1-HuACE2-H34K | HuACE2_H34K-F | ACAGGTCTTCGGCTTCCTTGTTAAACTTGTCCAAAAATGTCTTG |
|  |  | HuACE2_H34K-R | CAAGACATTTTTGGACAAGTTTAACAAGGAAGCCGAAGACCTGT |
| HuACE2 H34-L | pcDNA3.1-HuACE2-H34L | HuACE2_H34L-F | ACAGGTCTTCGGCTTCGAGGTTAAACTTGTCCAAA |
|  |  | HuACE2_H34L-R | TTTGGACAAGTTTAACCTCGAAGCCGAAGACCTGT |
| HuACE2 H34-Q | pcDNA3.1-HuACE2-H34Q | HuACE2_H34Q-F | AACAGGTCTTCGGCTTCCTGGTTAAACTTGTCCAA |
|  |  | HuACE2_H34Q-R | TTGGACAAGTTTAACCAGGAAGCCGAAGACCTGTT |
| HuACE2 H34-R | pcDNA3.1-HuACE2-H34R | HuACE2_H34R-F | ACAGGTCTTCGGCTTCGCGGTTAAACTTGTCCAAA |
|  |  | HuACE2_H34R-R | TTTGGACAAGTTTAACCGCGAAGCCGAAGACCTGT |
| HuACE2 H34-Y | pcDNA3.1-HuACE2-H34Y | HuACE2_H34Y-F | GGTCTTCGGCTTCGTAGTTAAACTTGTCCAAAAATGTCT |
|  |  | HuACE2_H34Y-R | AGACATTTTTGGACAAGTTTAACTACGAAGCCGAAGACC |
| HuACE2 D38-F | pcDNA3.1-HuACE2-D38F | HuACE2_D38F-F | AAGTGAACTTTGATAGAACAGGAATTCGGCTTCGTGGTTAAACTTG |
|  |  | HuACE2_D38F-R | CAAGTTTAACCACGAAGCCGAATTCCTGTTCTATCAAAGTTCACTT |
| HuACE2 D38-I | pcDNA3.1-HuACE2-D38I | HuACE2_D38I-F | AAGTGAACTTTGATAGAACAGGATTTCGGCTTCGTGGTTAAACTTG |
|  |  | HuACE2_D38I-R | CAAGTTTAACCACGAAGCCGAAATCCTGTTCTATCAAAGTTCACTT |
| HuACE2 D38-L | pcDNA3.1-HuACE2-D38L | HuACE2_D38L-F | GCAAGTGAACTTTGATAGAACAGTAGTTCGGCTTCGTGGTTAAACTTGTC |
|  |  | HuACE2_D38L-R | GACAAGTTTAACCACGAAGCCGAACTACTGTTCTATCAAAGTTCACTTGC |
| HuACE2 D38-V | pcDNA3.1-HuACE2-D38V | HuACE2_D38V-F | GAACTTTGATAGAACAGGACTTCGGCTTCGTGGTTAA |
|  |  | HuACE2_D38V-R | TTAACCACGAAGCCGAAGTCCTGTTCTATCAAAGTTC |
| HuACE2 K353-H | pcDNA3.1-HuACE2-K353H | HuACE2_K353H-F | GATCCTGAAGTCGCCATGCCCCAGGTCCCAAGC |
|  |  | HuACE2_K353H-R | GCTTGGGACCTGGGGCATGGCGACTTCAGGATC |

| **Species** | **NCBI Accession** | **Sequence homology** | **24** | **27** | **28** | **30*** | **31*** | **34*** | **35** | **37** | **38*** | **41** | **42** | **45** | **79** | **82** | **83** | **330** | **353*** | **354** | **355** | **357** | |
| --- | --- | --- | --- | --- | --- | --- | --- | --- | --- | --- | --- | --- | --- | --- | --- | --- | --- | --- | --- | --- | --- | --- | --- |
| Human | GQ262784 | 100.0% | Q | T | F | D | K | H | E | E | D | Y | Q | L | L | M | Y | N | K | G | D | R | |
| Cattle | BT021667 | 81.0% | Q | T | F | E | K | H | E | E | D | Y | Q | L | M | T | Y | N | K | G | D | R | |
| Cat | NM_001039456 | 85.2% | L | T | F | E | K | H | E | E | E | Y | Q | L | L | T | Y | N | K | G | D | R | |
| Civet | AY881174 | 83.5% | L | T | F | E | T | Y | E | Q | E | Y | Q | V | L | T | Y | N | K | G | D | R | |
| Dog | NM_001165260 | 83.5% | L | T | F | E | K | Y | E | E | E | Y | Q | L | L | T | Y | N | K | G | D | R | |
| Ferret | NM_001310190 | 82.6% | L | T | F | E | K | Y | E | E | E | Y | Q | L | H | T | Y | N | K | R | D | R | |
| Hamster | XP_005074266 | 84.5% | Q | T | F | D | K | Q | E | E | D | Y | Q | L | L | N | Y | N | K | G | D | R | |
| Pig | EU518378 | 81.4% | L | T | F | E | K | L | E | E | D | Y | Q | L | I | T | Y | N | K | G | D | R | |
| Mouse | BC026801 | 82.1% | N | T | F | N | N | Q | E | E | D | Y | Q | L | T | S | F | N | H | G | D | R | |
| Rat | GQ262788 | 82.5% | K | S | F | N | K | Q | E | E | D | Y | Q | L | I | N | F | N | H | G | D | R | |
| Potential critical residues of ACE2 for SARS-CoV-2 virus-contacting are indicated with an asterisk.  Amino acid changes in different animal species compared to the human ACE2 sequence are highlighted. | | | | | | | | | | | | | | | | | | | | | | |  |

**Table S3** Sequence alignment of ACE2 from human and diverse animal species.
